# Asymmetric Dynamics of the Native Aortic Annulus Evaluated by Force Transducer and Sonomicrometry in a Porcine Model

**DOI:** 10.1007/s13239-019-00418-1

**Published:** 2019-06-07

**Authors:** Leila Louise Benhassen, Diana Mathilde Ropcke, Troels Lading, Jens Kæstel Skov, Tommy Bechsgaard, Søren Nielsen Skov, Sten Lyager Nielsen, John Michael Hasenkam

**Affiliations:** 1 Department of Clinical Medicine, Aarhus University Hospital, Skejby, Aarhus, Denmark; 2 Department of Cardiothoracic and Vascular Surgery, Aarhus University Hospital, Aarhus, Denmark; 3 Department of Engineering, Faculty of Science and Technology, Aarhus University, Aarhus, Denmark

**Keywords:** Aortic annulus, Repair procedures, Geometrical characterization, Force distribution, Segmental variation, Expansibility

## Abstract

**Purpose:**

With new repair techniques of the aortic root and valve emerging, a detailed understanding of the dynamics of the aortic annulus and valve is required for optimal results. The objective of this study was to characterize geometrical changes and force distribution of the native porcine aortic annulus throughout the cardiac cycle.

**Methods:**

Measurements were performed in an acute 80 kg porcine model (*n* = 7) using sonomicrometry crystals in the aortic annulus for evaluation of geometry and dynamics, annular force transducer evaluating force distribution, and pressure measurements and echocardiography evaluating valve performance.

**Results:**

Overall, segmental force distribution and geometrical changes differed between different segments of the aortic annulus. The highest force development was found at the left/right interleaflet triangle (2.87 ± 2.1 N) and the largest segmental expansion was observed at the right-coronary and left-coronary sinus. The aortic annulus changed configuration throughout the cardiac cycle and became more oval in systole.

**Conclusions:**

This study is the first to describe detailed segmental dynamics and force distribution of the native aortic annulus in a porcine model *in vivo*. The heterogenous behavior of the aortic annulus suggests that different segments demand different support for repair of the aortic root and valve.

## Introduction

With the development of new surgical techniques for preservation and repair of the native aortic root and valve, a detailed understanding of the dynamics of the aortic annulus and valve is required. The aortic annulus plays an important role for the durability after aortic root repair, thus support and stabilization of the aortic annulus, while preserving the physiology of the native aortic root, is essential for good long-term results.^6^ The physiology and dynamics of the native aortic annulus is particularly important when performing aortic valve repair techniques, as the aim is to mimic and restore the properties of the native aortic annulus. The aortic annulus is heterogenous in composition and is comprised of the upper interventricular septum and the base of the anterior leaflet of the mitral valve. Because of the anatomic asymmetry, the dynamics and force distribution of the aortic annulus is similarly expected to be asymmetric throughout the cardiac cycle, but these aspects are not well understood. Only few studies have been performed with characterization of the aortic annulus as being oval in diastole and round in systole or primarily oval in both systole and diastole.^5^, ^11^ However, the segments in which the expansion primarily occur and how this affect the force distribution in the aortic annulus have not previously been described. Previous studies using sonomicrometry have been limited by using only three crystals in the aortic annulus, making it harder to gain detailed segmental geometrical data.^3^, ^4^, ^7^ A detailed anatomical description of the aortic annulus throughout the cardiac cycle is needed in order to understand this complex structure and its role in the dynamic behavior of the aortic root. A complete understanding of the native aortic annular biomechanics could potentially give important insight into which segments that have the highest demand for support when performing repair procedures of the aortic root and valve.

The aim of this *in vivo* study was to characterize and describe the geometry, dynamics and force distribution of the porcine native aortic annulus throughout the cardiac cycle by using detailed force-, geometry- and pressure measurements.

## Materials and Methods

A total of nine 80 kg pigs aged 6 months (Mixed Duroc and Landrace-Yorkshire) comprised the study material in this acute open-chest experimental study. Out of nine pigs, two pigs could not be weaned from extracorporeal circulation (ECC), which left a total of seven pigs for analysis. The study complied with Danish guidelines for experimental animal research. The study was approved by the Danish Inspectorate of Animal Experimentation.

### Force and Geometry Measurements

**Figure. 1:**
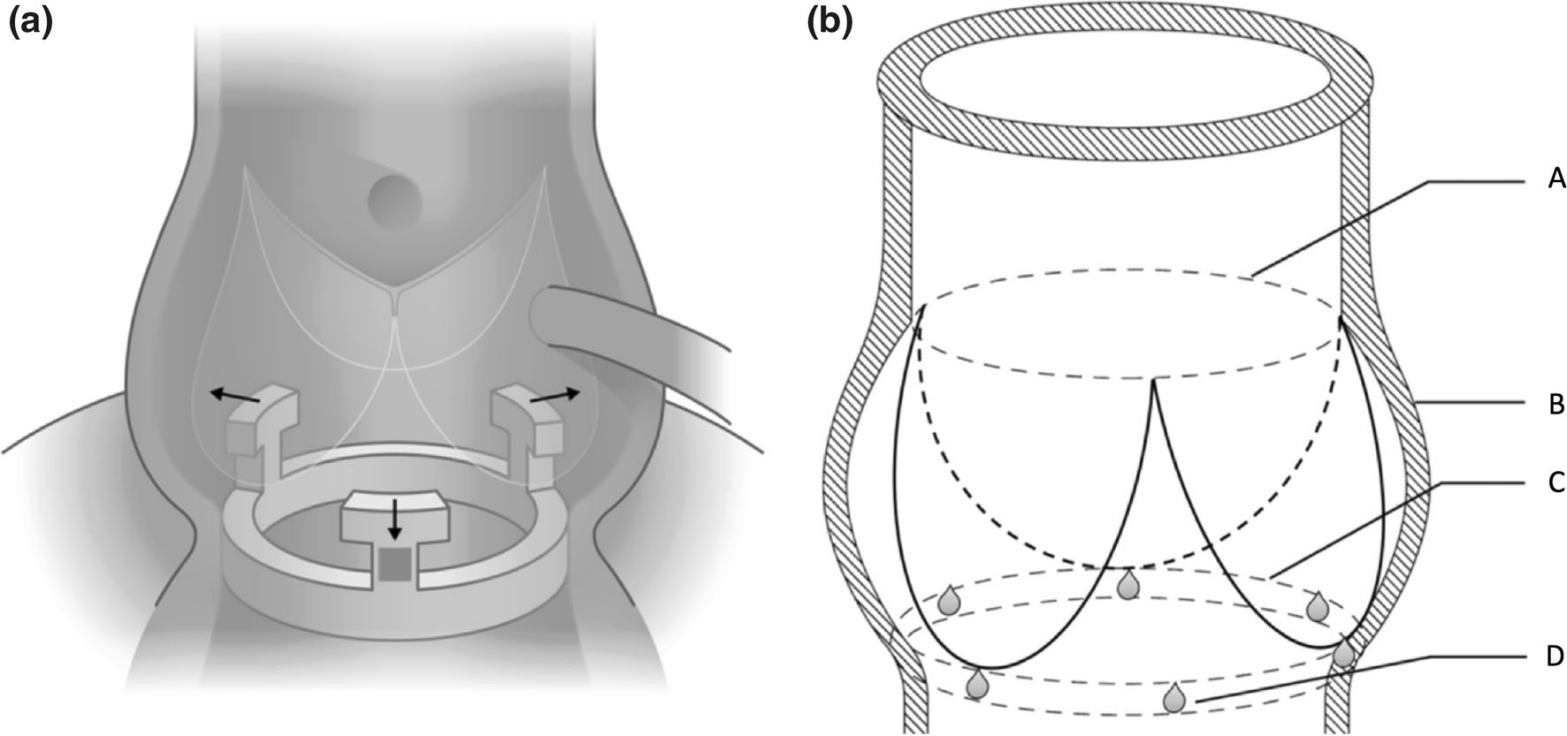
(a) Position of force transducer at the aortic annulus with three arms at each interleaflet triangle at annular level. Arrows indicate direction of force measurement during expansion. (b) Position of six sonomicrometry crystals at the aortic annulus. Reference crystals in the ascending aorta and apex of the left ventricle are not displayed. *A* Sinotubular junction, *B* sinuses of valsalva, *C* aortic annulus, *D* sonomicrometry crystal.

A force transducer with three measuring arms was implanted inside the aortic root at the level of the aortic annulus with each arm located upstream of the interleaflet triangle (Fig. 1), as previously described.^2^ The dedicated force transducer was developed for this study to measure radial forces in three individual segments corresponding to the three interleaflet triangles at the level of the aortic annulus: i.e., the left/right coronary (*LR*), right/non-coronary (*RN*) and left/non-coronary interleaflet triangle (*LN*). A similar force transducer design has previously been published from our group.^2^ However, this new force transducer design was enhanced by further reducing the frame of the transducer by making the transducer arms vertical instead of horizontal, thereby minimizing cross-talk and obstruction of the left ventricular outflow tract (Fig. 1). The transducer consisted of a basal ring and three equidistantly placed vertical arms with a diameter of 20 mm and 19 mm, respectively. On each arm, two strain gauges were attached to form a Wheatstone half-bridge. The force transducer measured strain corresponding to radial compression and distension of the aortic annulus. Prior to each experiment, the force transducer was calibrated to convert the output to force.

For geometrical measurements, a total of eight sonomicrometry crystals (Sonometrics Corp., London, Ontario, Canada) were implanted. The sonomicrometry method has been extensively validated and has been described in detail in earlier porcine studies.^3^, ^7^ Six 2 mm crystals were implanted at the level of the aortic annulus with one at each nadir of the leaflet and one at each interleaflet triangle at the tip of the transducer arms to get an anatomically correct segmental description of data (Fig. 1). Additionally, one crystal was implanted at the apex of the left ventricle and one crystal was implanted high in the ascending aorta as reference crystals.

### Surgical Protocol

Transportation, medication and handling of the animals have previously been described in detail.^8^, ^9^ Universal anesthesia was maintained with intravenously infusion of 3.7 mg/h/kg Propofol, 6.2 mcg/h/kg Fentanyl and 2.5 mg/h/kg Rocuronium. Heart rate, heart rhythm, invasive blood pressure, temperature and oxygen saturation were monitored continuously.

After sternotomy, cardiopulmonary bypass and cardioplegic arrest were established. The aortic valve was exposed through a transverse aortotomy approximately one cm downstream of the sinotubular junction. The sonomicrometry crystals were inserted through a small incision in the apex of the left ventricle. Each of the six crystals were fixated at each nadir of the leaflet and interleaflet triangle at annular level using 2-0 PremiCron (B. Braun, Melsungen, Germany^®^). Additionally, one crystal was inserted in the apex of the left ventricle, and one was inserted in the ascending aorta using 2-0 PremiCron. The force transducer was then inserted through the apical incision and sutured to the interleaflet triangles inside the aortic root at annular level using 2-0 PremiCron and secured using tourniquets placed outside the aorta. The aorta was closed using running 4-0 Prolene sutures. Mikro-Tip pressure catheters (SPR-350, Millar Instruments, Houston, TX, USA) were placed in the left ventricle through the apex and the ascending aorta. After reperfusion, weaning off cardiopulmonary bypass and hemo-dynamic stabilization, data collection was performed for 20 s, with collection of force-, pressure- and ECG data during breath hold. Following data acquisition, the force transducer was released and pulled down into the left ventricle. Hereafter, the second data collection was performed comprising of geometrical-, pressure- and ECG data without the force transducer *in situ* to avoid its impact on the geometrical measurements. Two-dimensional echocardiography (Vivid I, GE Vingmed Ultrasound AS, Horten, Norway) was performed to verify valve competence at baseline, following force transducer implantation, and after transducer removal. The animals were euthanized under continuous anesthesia with intravenous injection of an overdose of pentobarbital. The heart was excised and the position of the sonomicrometry crystals was confirmed. All animals were operated under standardized conditions by the same surgeon.

### Excluded Data

Due to malfunctioning, data from one sonomicrometry crystal was excluded from one pig and data from one transducer-arm was excluded from another pig.

### Data Acquisition and Data Analysis

The ventricular and aortic pressure were acquired using Mikro-Tip catheters and amplified with a pressure control unit (PCU-2000, Millar Instruments). Strain signals from the annular force transducer were obtained using data acquisition hardware (cDAQ model 9172 and NI-9237, National Instruments, Austin, TX, USA). The analogue data (pressure, force and ECG) were acquired with a sample rate of 1613 Hz using dedicated hardware and software (NI cDAQ 9172, NI 9237, NI 9215 and LabVIEW 2015, National Instruments, Austin Texas, USA). The sonomicrometry data was collected with a sample rate of 297 Hz using the Sonometrics TRX USB transceiver system and the SonoLabDS3 acquisition software package (Sonometrics Corp.). Ten cardiac cycles were used for analysis.

The first time derivate of the left ventricular pressure (LV-d*P*/d*t*) was used for synchronization between the analogue and sonomicrometry signals. *End*-*systole* was defined as d*P*/d*t* minimum and *End*-*diastole* was defined as the R peak in the ECG. *Mid*-*systole* was defined as the time point between d*P*/d*t* maximum and minimum and *Mid*-*diastole* was defined as the time point between End-systole and the following d*P*/d*t* maximum. The *Minimum* and *Maximum* time points were the amplitude minimum and maximum with respect to each single parameter reported. Two time points were calculated; from Mid-diastole to Mid-systole (Mid-systole-Mid-diastole), and from Minimum to Maximum (Maximum-Minimum). All six segmental distances were used for analysis. Furthermore, we calculated three anatomically correct segmental distances, i.e., the length of the non-coronary sinus (NC), right coronary sinus (RC) and left coronary sinus (LC). For analysis of the shape of the aortic annulus, three anatomical cross-sectional diameters were calculated, that corresponded to the sinus-commissure diameter for each segment throughout the cardiac cycle, i.e., NC-LR, RC-LN, LC-RN. The difference between the smallest and largest cross-sectional diameter was calculated. To evaluate the shape of the aortic annulus, the definition by Tops *et al.* was used,^12^ which defined the aortic annulus as oval if the difference between two cross-sectional diameters was greater than 3 mm. Geometrical data was reported as the amplitude difference between Maximum and Minimum values and the cross-sectional areas as the amplitude difference between Mid-systole-Mid-diastole to evaluate the shape of the aortic annulus throughout the cardiac cycle. Deformational force data was reported as the amplitude difference between Maximum and Minimum values.

### Statistical Analysis

All data are presented as mean ± SD from ten consecutive heart cycles with a significance level of *p* < 0.05. The collected data were analyzed by two-way repeated measures ANOVA using anatomical segment and repetitive heart cycles as factors. The model allowed for different residual variations. Anatomical segments were then compared using *post hoc* Wald *z*-tests. Residuals were inspected for normality and no reason to refute this was found. The data were analyzed using Stata 13.0 (StataCorp LLC, Texas, USA). Development of the statistical models were performed with support from Aarhus University (BIAS, University of Aarhus, Aarhus, Denmark).

## Results

Hemodynamic parameters are presented in Table 1. Total ECC time was 183 ± 20 min and cross clamp time was 114 ± 13 min. The transvalvular pressure loss across the aortic valve did not reveal any significant difference with or without the force transducer *in situ* (24 ± 7 vs. 23 ± 14 mmHg, *p* = 0.108). There was a significantly higher left ventricular pressure after force transducer removal compared with before removal. All pigs had no or trivial aortic regurgitation on epicardial echocardiography at baseline, following force transducer implantation, and after transducer removal. The results presented in the manuscript, were obtained during the hemodynamic conditions listed in Table 1.

**Table 1:** Hemodynamic parameters before and after the procedure.

	HR (min^−1^)	LVP maximum (mmHg)	Max transvalvular pressure loss (mmHg)
With transducer	93 ± 15	93 ± 20	23 ± 14
Without transducer	96 ± 18	101 ± 11	24 ± 7
*P* value	0.765	0.008	0.108

Mean ± SD. *HR* Heart rate, *LVP* left ventricular pressure

### Force Results

**Figure. 2:**
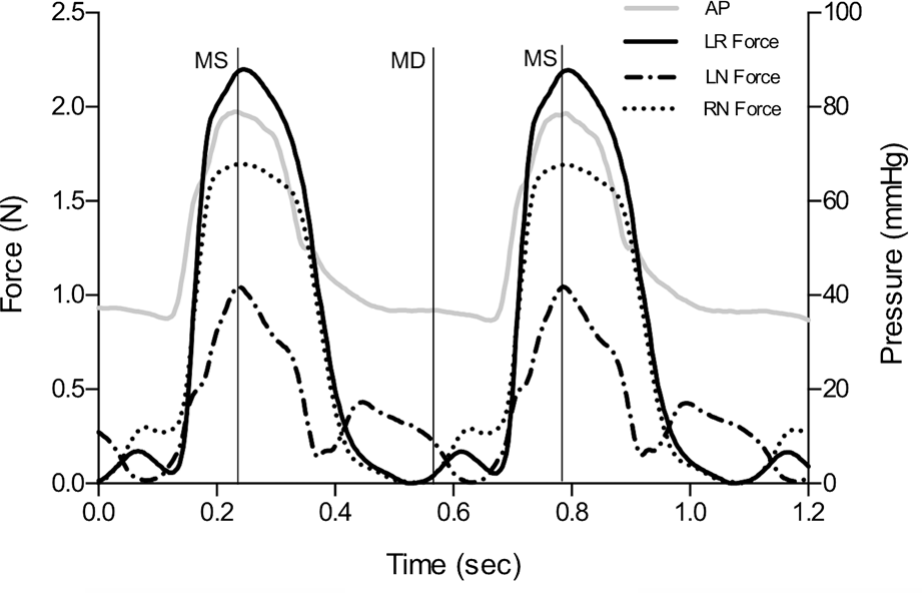
Relationship between force at the three interleaflet triangles of the aortic annulus (LN, RN, LR) and aortic pressure from one representative pig throughout two cardiac cycles. The force measurements offsets were zeroed in mid-diastole. *AP* Aortic pressure, *MS* mid-systole, *MD* mid-diastole, *LN* left/non-coronary interleaflet triangle, *RN* right/non-coronary interleaflet triangle, *LR* left/right interleaflet triangle.

**Figure. 3:**
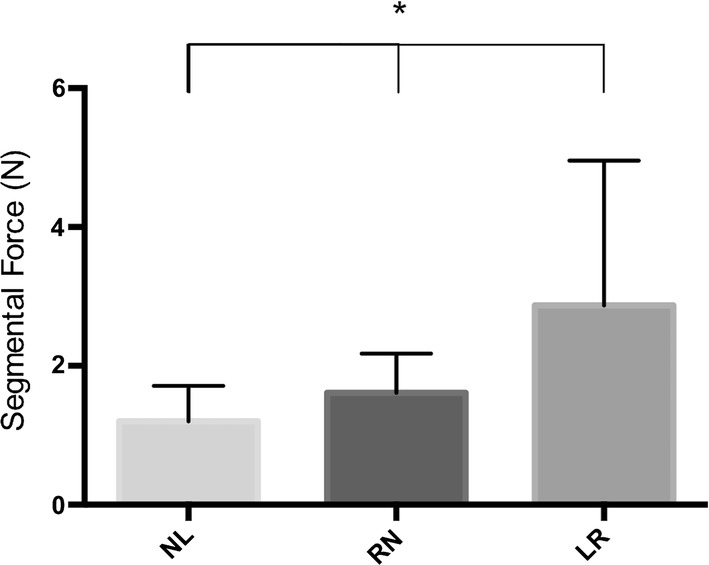
Maximum–Minimum radial force for each of the three interleaflet triangles of the aortic annulus (LN, RN, LR). *LN* Left/non-coronary interleaflet triangle, *RN* right/non-coronary interleaflet triangle, *LR* left/right interleaflet triangle. Mean + SD. *p < 0.05 between all three segments.

In Fig. 2, force- and aortic pressure measurements from one representative pig are depicted. The segmental forces from Minimum to Maximum are presented in Fig. 3. The force development was significantly different in all three segments being highest at LR from Minimum to Maximum (LN 1.20 ± 0.51 N; RN 1.61 ± 0.56 N; LR 2.87 ± 2.08 N, *p* < 0.05). The force pattern revealed that the force at LR was highest near Mid-systole and lowest at Mid-diastole. However, at LN and RN, the force peaked later and was highest between Mid-systole and End-systole and lowest between Mid-diastole and End-diastole.

### Geometrical Results

**Figure. 4:**
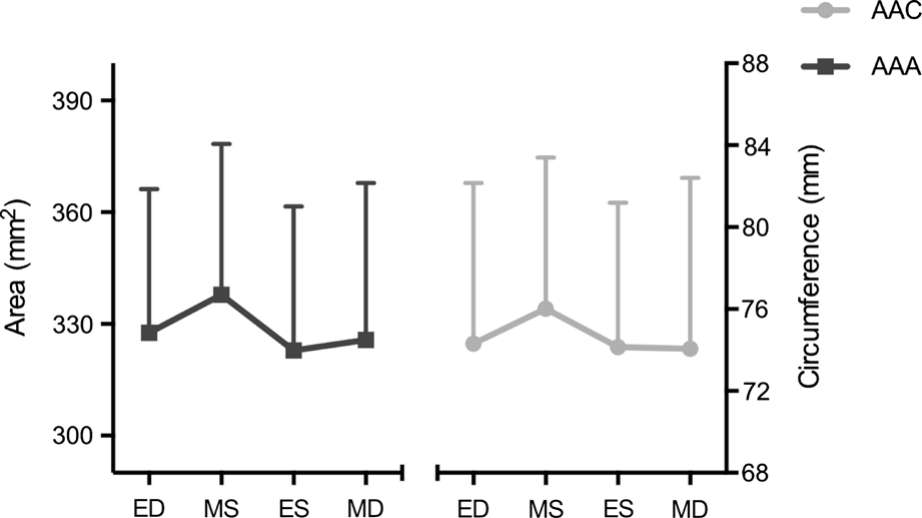
Aortic annulus area (AAA) (left) and aortic annulus circumference (AAC) (right) presented at four defined time points throughout the cardiac cycle (ED, MS, ES, MD). *ED* End-diastole, *MS* mid-systole, *ES* end-systole, *MD* mid-diastole. Mean + SD.

**Figure. 5:**
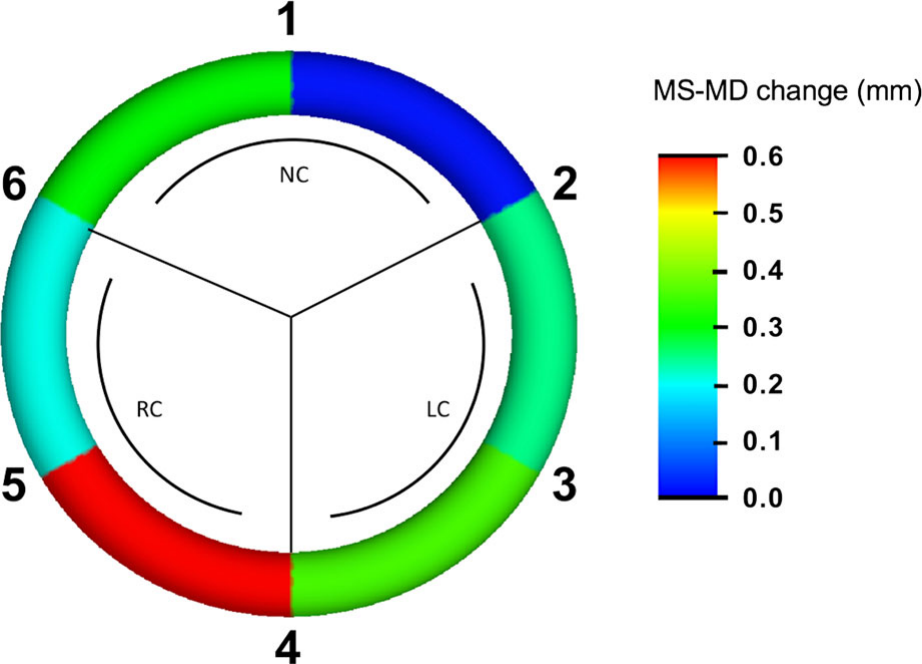
Annular segmental expansion from Mid-diastole to Mid-systole (MS-MD) illustrated with a scaled color legend. Red indicates a large expansion and blue indicates no expansion. *NC* Non-coronary sinus, *LC* left coronary sinus, *RC* right coronary sinus.

Geometrical results are summarized in Table 2. The geometrical results for aortic annulus area (AAA) and aortic annulus circumference (AAC) are presented in Fig. 4 as Maximum-Minimum values and at four defined time points (End-systole, Mid-systole, End-diastole, Mid-diastole) throughout the cardiac cycle. There was a significant difference between Minimum and Maximum AAA with an expansion of 12 ± 7% (*p* < 0.005). Also, we found a significant difference between Minimum and Maximum AAC with an expansion of 6 ± 2% (*p* < 0.005). Change in segment length from Mid-diastole to Mid-systole for each of the six annular segments is illustrated with a scaled color legend as an expression of the segmental expansion in Fig. 5 and in Table 2. The largest expansion was observed at the RC and the smallest at the NC from Minimum to Maximum (NC: 1.7 ± 0.6 mm; LC 1.9 ± 1.0 mm; RC 2.5 ± 1.3 mm, *p* < 0.05).

**Table 2:** Aortic annulus geometry and dynamics.

Parameter	Maximum	Minimum	Change	*p* value
AAA (mm^2^)	346 ± 40	311 ± 45	35 ± 16	<0.001
AAC (mm)	76.9 ± 8.0	72.5 ± 8.0	4.4 ± 1.5	<0.001
Segmental distances				
NC (mm)	21.4 ± 6.7	19.7 ± 6.4	1.7 ± 0.6	<0.001
RC (mm)	29.2 ± 4.8	26.7 ± 4.3	2.5 ± 1.3	<0.001
LC (mm)	27.4 ± 6.0	25.5 ± 5.5	1.9 ± 1.0	<0.001

Mean ± SD. *AAA* Aortic annulus area, *AAC* aortic annulus circumference, *NC* non-coronary sinus, *RC* right coronary sinus, *LC* left coronary sinus

**Figure. 6:**
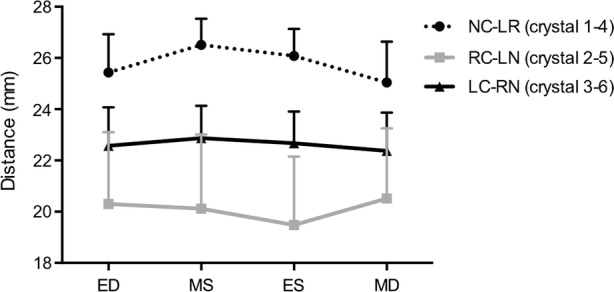
Cross-sectional diameters NC–LR (crystal 1–4), RC–LN (crystal 2–5) and LC–RN (crystal 3–6) presented at four defined time points throughout the cardiac cycle. *ED* End-diastole, *MS* mid-systole, *ES* end-systole, *MD* mid-diastole, *NC* non-coronary sinus, *LR* left/right interleaflet triangle, *RC* right coronary sinus, *LN* left/non-coronary interleaflet triangle, *LC* left coronary sinus, *RN* right/non-coronary interleaflet triangle. Mean + SD.

The cross-sectional diameters are presented in Fig. 6 at four defined time points throughout the cardiac cycle. The largest cross-sectional diameter was observed for segment 1–4 in both Mid-systole and Mid-diastole (Mid-systole: 26.5 ± 1.0 mm; Mid-diastole: 25.0 ± 1.6 mm). The smallest cross-sectional diameter was for segment 2–5 at both Mid-systole and Mid-diastole (Mid-systole: 20.1 ± 2.9 mm; Mid-diastole: 20.5 ± 2.8 mm). The largest expansion was observed between segment 1–4 from Mid-diastole to Mid-systole with a mean expansion of 1.5 ± 0.9 mm. Segment 2–5 and 3–6 did not reveal any significant pattern of movement from Mid-diastole to Mid-systole, and we found an expansion in some pigs and a contraction in others. We found that the aortic annulus was oval at Mid-systole and became more round at Mid-diastole in all pigs.

## Discussion

In this acute porcine open-chest model, we evaluated the dynamics of the aortic annulus with segmental force- and geometrical measurements. The model was successful for the intended characterization with a dedicated force transducer to evaluate force distribution, and sonomicrometry crystals to measure geometrical changes throughout the cardiac cycle.

This study is the first to describe the force distribution in the aortic annulus in combination with detailed geometrical changes with high spatial resolution and sampling rate by using six sonomicrometry crystals in the aortic annulus. Furthermore, this study is the first to evaluate the shape of the aortic annulus in a porcine model by accurate anatomical landmarks throughout the cardiac cycle.

The transvalvular pressure loss did not reveal any significant change before and after force transducer implantation. The force transducer was designed to minimize obstruction in the left ventricular outflow tract, which was confirmed by the low transvalvular pressure loss across the aortic valve with the transducer *in situ*. At both Mid-systole and Mid-diastole, the segment with the maximum force was the LR, which is the muscular segment of the aortic annulus. The smallest force was at LN, which corresponds to the fibrous segment of the aortic annulus, i.e., the anterior leaflet of the mitral valve. This finding is in accordance with previous findings from our group,^3^, ^10^ where force in LR was found to be significantly higher than the two other segments. Our group previously evaluated the aortic annulus from a pig heart in a symmetric pulsatile *in vitro* model, where we found the same force pattern, suggesting that the heterogenous tissue composition in the aortic annulus plays an important role in force development.^10^ Furthermore, we found a delay in peak force between LR and the two other segments. This delay in force development could be explained by the asymmetric hemodynamic ejection which is caused by contraction and torsion of the left ventricular outflow tract. The force pattern reveals a heterogenous stress distribution of the native aortic annulus with the highest stress being in the LR. These results suggest that the LR segment has a higher need for support and stabilization when performing aortic valve repair procedures to avoid redilatation and achieve good long-term results.

The geometrical measurements showed that there was a significant increase in AAA and AAC throughout the cardiac cycle of 12 and 6%, respectively. These are both within the normal physiological range for adult patients and in accordance with previous studies on the human aortic root.^1^ Previous studies of the geometry of the aortic annulus have been limited by only three sonomicrometry crystals in the aortic annulus, thus the calculated area of the aortic annulus was not accurate.^3^, ^7^ We found that the largest circumferential expansion from Mid-diastole to Mid-systole was between the right and left sinus, which is the only segment primarily consisting of muscle fibers, and thus being able to contract. Lansac *et al*.^7^ investigated the aortic root in eight sheep, and found the same heterogenous pattern of expansion with the largest expansion being between the left–right sinus. No circumferential expansion was observed between the NC–LN segment, which is a segment dominated by fibrous fibers from the mitral valve.

The largest cross-sectional diameter was observed between LR–NC in Mid-systole and Mid-diastole and the largest radial expansion was also observed here. We found that the aortic annulus was oval in Mid-systole and became rounder in diastole. With the definition used by Tops *et al.*,^12^ all pigs had an oval aortic annulus in Mid-systole and only 50% of the pigs had an oval aortic annulus in Mid-diastole. However, the aortic root in pigs is slightly smaller than in human adults, thus a difference of 3 mm between two cross-sectional diameters in pigs would represent a more pronounced oval shape than in humans. Nonetheless, all pigs had a more oval shaped aortic annulus in systole compared with diastole. This finding is contrary to previous findings in humans.^5^, ^11^ Sucha *et al.*^11^ investigated the aortic annulus from two perpendicular cross-sectional diameters that were not anatomically defined. They found that the short diameter axis significantly changed in dimension, whereas the maximal diameter remained relatively unchanged, resulting in an oval shaped annulus in diastole and round shaped annulus in systole. However, the definition of sphericity is not specified, the evaluation of the aortic annulus is based on both echocardiography and CT scans and the patient population consisted of both healthy adult individuals and patients with aortic valve stenosis. De Heer *et al.*^5^ investigated the deformation of the aortic annulus in healthy adult individuals and concluded that the aortic annulus was oval in both systole and diastole (defined by > 3 mm difference between minimum and maximum cross-sectional diameter) in most patients. In that study the annulus was evaluated by computed tomography but was only compared by two perpendicular cross-sectional diameters that were not anatomically defined. Therefore, the roundness of the aortic annulus would be overestimated if the annulus was oval in an oblique direction to the two planes. We investigated the geometrical changes of the aortic annulus throughout the cardiac cycle by three anatomically correct cross-sectional diameters. The finding of a predominantly oval aortic annulus in the pig, but also that the long-axis was found between LR–NC is new knowledge and should be confirmed by additional studies in humans.

Both force- and geometrical data showed that the segment between the left and right sinus had the highest force development and largest circumferential and radial expansion compared with the other segments. These results imply that this segment might be more vulnerable to re-expand compared with other parts of the aortic annulus due to the higher stress load and expansion of this segment. The heterogenous dynamics and force distribution in the native aortic annulus suggest that each segment of the aortic annulus demands different support to avoid re-dilatation but still preserve the dynamics of the native aortic root. The asymmetric behavior of the aortic annulus should be considered, and the segment at the right and left sinus could have a higher demand for support and stabilization when performing repair procedures of the aortic root and valve.

These measurements of the native aortic annulus in the porcine model might serve as reference for future studies investigating repair techniques and the development of new medical devices for the native aortic root and valve.

## Study Limitations

Because of the high degree of invasiveness in this method, it was not possible to perform in humans. This study was performed in porcine hearts, which has many similarities to the human heart, but also differs in some ways; the right coronary sinus of pig hearts has a wide septal muscle shelf just below the insertion of the right cusp, which could potentially change the geometry and force in the aortic annulus. However, the porcine model is an acknowledged and anatomically comparable model for experimental research of this caliber. This study was an acute invasive, open chest study, and the position of the sonomicrometry crystals and force transducer could also be expected to affect the mechanical properties and outflow tract in some way. Nonetheless, there was no increase in transvalvular pressure across the aortic valve, thus it did not create an aortic valve stenosis. Furthermore, the transducer design was enhanced to minimize cross-talk and obstruction of the left ventricular outflow tract. Other options of imaging modalities are magnetic resonance imaging, computed tomography (CT) and echocardiography. However, these methods are limited by lower spatial resolution and sampling rate and the anatomical landmarks are less well-defined.
